# [Expression of Concern] Obesity accelerates murine gastric cancer growth by modulating the Sirt1/YAP pathway

**DOI:** 10.3892/ol.2026.15811

**Published:** 2026-08-12

**Authors:** Hai-Jun Li, Jun-Ke Fu, Xiang-Ming Che, Lin Fan, Yong Zhang, E Bai

Oncol Lett 14: 4151–4157, 2017; DOI: 10.3892/ol.2017.6715

Following the publication of the above paper, it has been drawn to the Editor's attention by a concerned reader that, regarding the results of the Sirt1 and YAP protein expression experiments shown in Figs. 1A and 2A respectively on p. 4155 (representing data from the obese mice in these experiments), the data panels portrayed contained an overlapping section, suggesting that these panels, which were intended to show the results of differently performed experiments, had apparently been derived from the same original source.

The authors have been contacted by the Editorial Office to offer an explanation for this apparent anomaly in the presentation of the data in this paper, and we are awaiting their response. Owing to the fact that the Editorial Office has been made aware of potential issues surrounding the scientific integrity of this paper, we are issuing an Expression of Concern to notify readers of this potential problem while the Editorial Office continues to investigate this matter further.

